# Children do not exhibit ambiguity aversion despite intact familiarity bias

**DOI:** 10.3389/fpsyg.2014.01519

**Published:** 2015-01-05

**Authors:** Rosa Li, Elizabeth M. Brannon, Scott A. Huettel

**Affiliations:** ^1^Department of Psychology and Neuroscience, Duke UniversityDurham, NC, USA; ^2^Center for Cognitive Neuroscience, Duke UniversityDurham, NC, USA; ^3^Center for Interdisciplinary Decision Sciences, Duke UniversityDurham, NC, USA; ^4^Brain Imaging and Analysis Center, Duke UniversityDurham, NC, USA

**Keywords:** ambiguity, risk, uncertainty, decision-making, ambiguity aversion, development, children, familiarity bias

## Abstract

The phenomenon of ambiguity aversion, in which risky gambles with known probabilities are preferred over ambiguous gambles with unknown probabilities, has been thoroughly documented in adults but never measured in children. Here, we use two distinct tasks to investigate ambiguity preferences of children (8- to 9-year-olds) and a comparison group of adults (19- to 27-year-olds). Across three separate measures, we found evidence for significant ambiguity aversion in adults but not in children and for greater ambiguity aversion in adults compared to children. As ambiguity aversion in adults has been theorized to result from a preference to bet on the known and avoid the unfamiliar, we separately measured familiarity bias and found that children, like adults, are biased towards the familiar. Our findings indicate that ambiguity aversion emerges across the course of development between childhood and adolescence, while a familiarity bias is already present in childhood.

## INTRODUCTION

Economists and psychologists distinguish between two types of decision-making under uncertainty: *risky* decisions feature uncertain outcomes with known probabilities (i.e., a 50% chance of winning $5), while *ambiguous* decisions feature uncertain outcomes with unknown probabilities (i.e., an unknown chance of winning $5; Knight, 1921; Ellsberg, 1961). Notably, while decision makers are averse to both types of uncertainty, decision makers tend to be even more averse to ambiguity than they are to risk – at least when tested as adults. For example, when offered the chance to win a prize by drawing a red ball from a risky urn of 50% red, 50% black balls or an ambiguous urn with an unknown mix of red and black balls, adults prefer to draw from the risky urn (Ellsberg, 1961). Adults are also willing to pay more for the chance to draw from a risky urn than from an ambiguous urn (Eisenberger and Weber, 1995). Ambiguity aversion has been consistently found in studies of adult decision-making behavior (see Camerer and Weber, 1992 for review) – and it shares at least some similarities with risk aversion (Lauriola et al., 2007).

Evidence suggests, however, that risk and ambiguity engage distinct processes as well (Lauriola et al., 2007). Adults’ behavioral risk preferences have been found to be correlated with their ambiguity preferences only under certain conditions (Lauriola and Levin, 2001) or not at all (Hogarth and Einhorn, 1990; Levy et al., 2010). Studies using neuroimaging techniques have found risky and ambiguous decision-making to share some neural processes in reward-related brain regions (Levy et al., 2010) but also to engage distinct circuitry elsewhere in the brain (Hsu et al., 2005; Huettel et al., 2006; Krain et al., 2006; Bach et al., 2009). Because assessments of risk and ambiguity have been found to be both behaviorally and neurally distinct, a complete understanding of decision-making under uncertainty must include both risky and ambiguous decision-making.

Several studies have investigated the development of risk preferences in children (Harbaugh et al., 2002; Levin and Hart, 2003; Eshel et al., 2007; Levin et al., 2007a,b; Rakow and Rahim, 2010; Paulsen et al., 2011, 2012; Weller et al., 2011) with the ultimate goal to predict and prevent real-world maladaptive reckless behavior. Decisions made outside of the laboratory, however, are more likely to be ambiguous than risky, as individuals rarely know the exact probability contingencies of such decisions. Because preferences for ambiguity may better predict real-world decision-making than do preferences for risk, an understanding of the development of ambiguity preferences is important for guiding policies to promote advantageous decision-making. Yet there is a paucity of studies investigating how ambiguity aversion emerges and changes across development. Just one study has compared adolescents (12- to 17-year-olds) to adults and found reduced ambiguity aversion in adolescents relative to adults in the gain domain (Tymula et al., 2012) but similar levels of ambiguity neutrality in adolescents and adults in the loss domain (Tymula et al., 2013). Another study found ambiguity aversion in adolescents (10- to 18-year-olds) but did not include an adult comparison group (Sutter et al., 2013). To the best of our knowledge, there have been no prior studies comparing ambiguity aversion in children to that of adults.

In the current study, we characterized attitudes towards risk and ambiguity in 8- and 9-year-old children and a comparison group of 19- to 27-year-old adults. Were children to treat ambiguity in the same manner as adults, we would find ambiguity aversion in both age groups. Instead, we found no evidence for ambiguity aversion in children, significant evidence for ambiguity aversion in adults, and significantly greater ambiguity aversion in adults than in children. Because different methods for measuring preferences can elicit inconsistent valuations of the same gamble (Lichtenstein and Slovic, 1971), we used multiple tasks in order to minimize the chance that our conclusions were specific to a particular task. Our results were consistent across multiple tasks and independent measures, indicating that our findings were robust to different methods of preference measurement. We additionally evaluated whether differences in ambiguity aversion between children and adults might, instead, reflect a relative preference for familiar stimuli – as advanced as a potential explanation for ambiguity aversion in adults (Heath and Tversky, 1991; Fox and Tversky, 1995; Fox and Weber, 2002). We measured children’s preference for betting on items that provided an illusion of greater knowledge in a child-friendly familiarity bias task, and found that children (like adults) exhibited a significant familiarity bias. Our findings indicate that ambiguity aversion emerges over the course of development from childhood to adulthood but disconfirm the alternative explanation that this emergence results from a delayed familiarity bias.

## MATERIALS AND METHODS

### PARTICIPANTS

Forty-two children (21 female; mean age = 8.7 years; range = 8.1–9.9 years) and 40 young adults (17 female; mean age = 22.4 years; range = 19.2–27.8 years) were recruited from the Raleigh-Durham-Chapel Hill area of North Carolina. We chose children of this age range because they are the youngest age that could comprehend all task instructions, are just beginning to receive formal education in fractions (Common Core State Standards Initiative [CCSSI], 2014), and are starting to make complex, accurate assessments of probability (Falk et al., 2012). Informed consent was collected from adult participants and parents of child participants, and written assent was collected from child participants under a protocol approved by the Institutional Review Board of Duke University. Children’s parents were paid $10 for their child’s participation, plus $10-15 for travel expenses. Child participants received toys of their choice. Adult participants were paid $10, plus a cash bonus that was based on the outcome of a randomly selected Bar Choice trial, a randomly selected willingness to pay (WTP) trial, and their accuracy on the Familiarity Bias task (see below).

### BAR STIMULI AND TRAINING

Participants were informed that they would be playing games to win tokens, with the goal to win as many tokens as possible. Children were informed that the tokens could be used to purchase prizes (toys and stickers) at the end of the study, while adults were informed that the tokens would be exchanged for a cash bonus at the end of the study. In order to minimize differences in subjective reward valuation between participants or age groups (Geier and Luna, 2012), the exchange rates for the tokens were not revealed to participants until the end of the experimental session. Children’s exchange rates were set on an individual basis so that all children could “purchase” one high quality toy or two medium quality toys. The exchange rate for adults was $0.25 per token.

**FIGURE 1:**
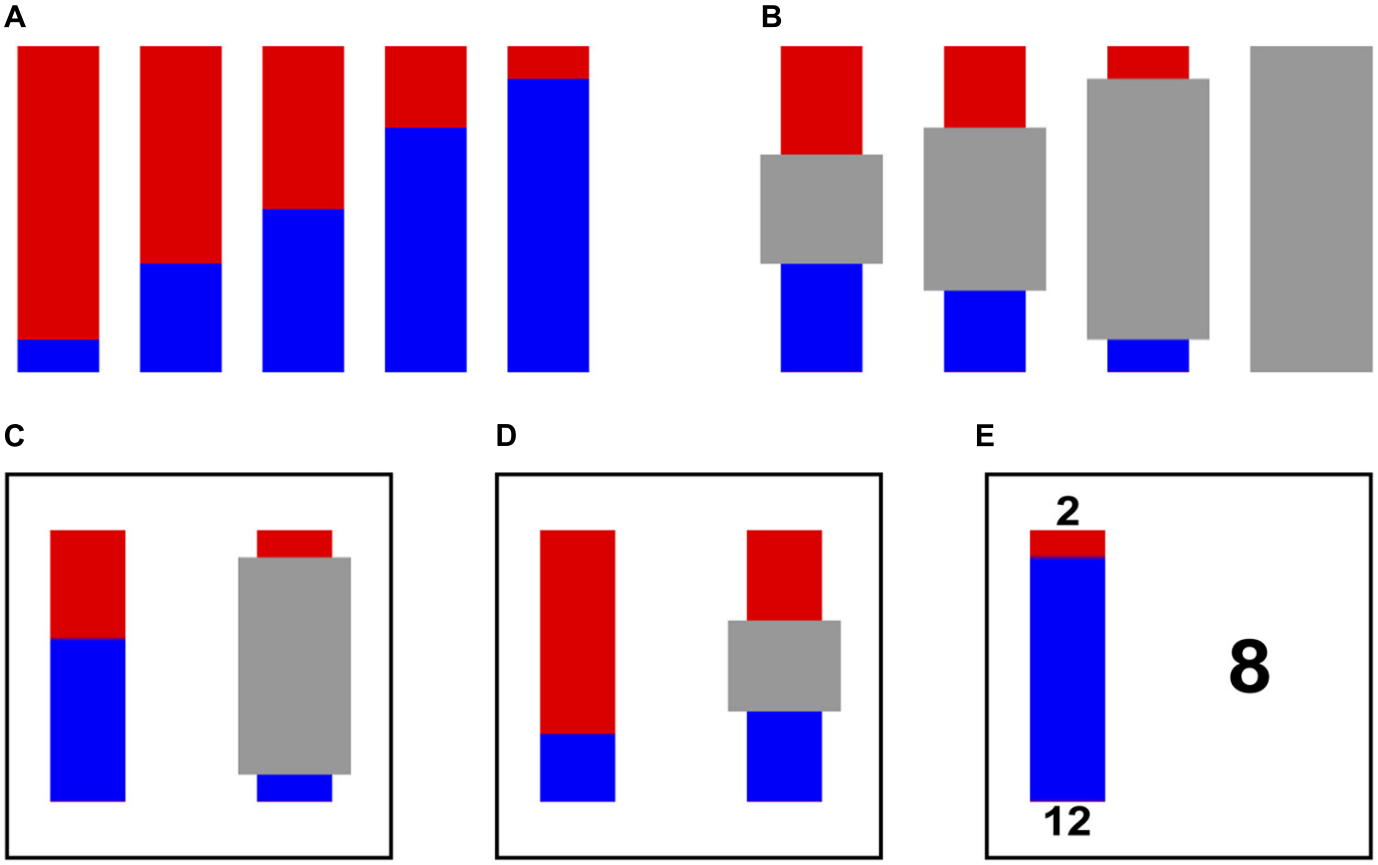
**Stimuli and tasks.** Red and blue represent the chance of winning a small (two tokens) or large (12 tokens) reward. The color representing the large reward was counterbalanced across participants. **(A)** Example risky bars. A total of 11 different risky bars representing different probabilities were used. **(B)** All four ambiguous bars used. **(C)** Example Bar Choice trial featuring a risky bar versus an ambiguous bar. Participants indicated with a key press which of the two bars they preferred. **(D)** Example of a Bar Choice “catch” trial. If red represented the bigger win, participants should select the risky bar. If blue represented the bigger win, participants should select the ambiguous bar. **(E)** Example willingness to pay (WTP) trial. Each bar was presented on the left of the screen with its endpoints labeled with their associated reward values. Participants used arrow keys to toggle the number on the right up or down until it reached their maximum WTP for the displayed bar.

Stimuli consisted of bars divided into red and blue portions (Hayden et al., 2010; Levy et al., 2010), in which the colors represented the chance of winning a small (2 tokens) or large (12 tokens) reward. The color representing the large reward was counterbalanced across participants. The red portion of the bars always appeared above the blue portion. Eleven risky stimuli (**Figure 1A**) representing 10, 25, 33, 40, 45, 50, 55, 60, 67, 75, and 90% chances of winning were used with all of the adults and 32 of the children (10 children used a subset of seven risky stimuli representing 10, 25, 33, 50, 67, 75, and 90% chances of winning; after initial testing in those 10 children revealed drastic preference shifts between 33 and 50% or between 50 and 67% chances of winning, additional levels of risk were added to capture more subtle changes in preference). Ambiguous stimuli (**Figure 1B**) featured a gray occluder centered at the midpoint of a risky bar. The size of the occluder varied across trials to determine the level of ambiguity (33, 50, 80, and 100%). The ambiguous bars revealed equivalent amounts of red and blue at the endpoints.

All participants were trained in the experimental stimuli and tasks before data collection. Children were trained one-on-one by an experienced experimenter. Adults read the instructions independently and then completed training trials under an experimenter’s supervision. The reward value (2 or 12 tokens) represented by the bars’ colors (red and blue) was explicitly stated to participants. The ambiguous bars were explained via animations in which the occluder moved laterally off a 50% ambiguous bar to reveal what colors were beneath it. All different combinations of red and blue (i.e., all red, all blue, half red and half blue, more red than blue, and more blue than red) were shown to underscore that the occluder could be hiding any probability. Both children and adults had to correctly answer questions about the stimuli and procedures before they were allowed to begin the experimental session. For example, to demonstrate their understanding of the risky stimuli, participants had to correctly explain which of several risky bars represented the greatest chance of winning the most tokens. To demonstrate their understanding of the ambiguous stimuli, participants had to correctly explain which risky bars could and could not be under an ambiguous bar’s gray occluder. Children were asked and answered the questions verbally while adults answered the same questions on a worksheet. If participants did not answer a question correctly, the experimenter repeated the training instructions until the participants could answer the question correctly. Average training time was approximately 15 min for children and 10 min for adults.

### TASKS

Participants performed four tasks in the following order: (1) Bar Choice (approximately 15 min), (2) WTP (approximately 10 min), (3) Bar Probability (approximately 5 min), and (4) Familiarity Bias (approximately 5 min). Total session time was approximately 50 min for children and 45 min for adults.

#### Bar Choice

In the Bar Choice task, participants were asked to choose which of two bars they preferred (**Figure 1C**). Participants were shown all possible pairings of risky versus ambiguous bars (28 trials for the 10 children who used the limited set of risky stimuli; 44 trials for all other participants) and ambiguous versus ambiguous bars (six trials for all participants). The bars remained on the screen until participants indicated their preference using a left or right key press. After a key press was made, a box highlighted the chosen bar, and participants were given the opportunity to change their response. A second key press was required to confirm the highlighted choice in order to minimize impulsive responding. The inter-trial-interval was 1-s.

Left and right positions of the risky and ambiguous bars were counterbalanced across trials. The order of trials was randomized across participants. Participants received no outcome feedback during the task. Instead, they were told that one trial would be selected at random at the end of the session, and they would be paid according to the outcome of their selected bar on that trial.

Six of the risky versus ambiguous trials served as “catch” trials because they featured a choice with an objectively correct answer. On these trials the switch from red to blue in the risky bar occurred within the portion of the bar that was not occluded in the ambiguous bar (**Figure 1D**; 10% win-33% ambiguous, 10% win-50% ambiguous, 90% win-33% ambiguous, 90% win-50% ambiguous, 25% win-33% ambiguous, and 75% win-33% ambiguous). The risky bar therefore clearly contained either a greater or smaller amount of the winning color than the ambiguous bar. These catch trials served as exclusion criteria for the Bar Choice task: a participant was excluded from analyzes if s/he missed one or more of these “catch” trials. Catch trials were excluded from reported results and analyzes.

Data from seven children and one adult were excluded from the Bar Choice task on the basis of these criteria, leaving a final Bar Choice sample of 35 children (17 female; mean age = 8.7 years) and 39 adults (16 female; mean age = 22.4 years).

#### Willingness to Pay (WTP)

In the WTP task, participants were endowed with 12 tokens. They were then shown each risky and ambiguous bar with the red and blue ends labeled with their respective reward values. Participants were asked to press arrow keys to toggle a number up or down until the number reached their maximum WTP for the chance to play that bar (**Figure 1E**). An additional key press confirmed the WTP value selection.

The order of trials was randomized across participants, and each trial’s WTP start value was randomized to start at 2 or 12. Participants were informed that one trial would be selected at random at the end of the session and a price would be randomly selected for that trial. If participants’ WTP was greater than or equal to the randomly selected price, they would pay that price from their endowment to “buy the bar” and then be paid according to the outcome of their purchased bar. If their WTP was less than the randomly selected price, they would pay nothing and receive nothing (Becker et al., 1964).

Data from the seven children and 1 adult whose data were excluded from Bar Choice task for failing to understand the bar stimuli, as noted above in *Bar Choice*, were also excluded from WTP. Data from an additional child was excluded from WTP because her WTP for the risky bars did not increase with the probability of winning, indicating a failure to understand the WTP task. All other participants’ WTP for the risky bars increased with the probability of winning, leaving a final WTP sample of 34 children (16 female; mean age = 8.7 years) and 39 adults (16 female; mean age = 22.4 years).

#### Bar Probability

In the Bar Probability task, participants were shown each risky and ambiguous bar and were asked to predict how many times the bar would result in red and blue across 100 hypothetical trials. Children verbally gave their responses to an experimenter, while adults indicated their responses on a worksheet.

One child who struggled to produce responses on a scale of 100 trials was asked to give responses out of 10 hypothetical trials. Four children who struggled to produce responses that summed to 100 trials were permitted to indicate their response for just one color of their choosing rather than for both colors. These modifications allowed children with limited mathematical skills to perform the task while reducing frustrations.

#### Familiarity Bias

In order to measure familiarity bias, participants were given the chance to win additional tokens by correctly guessing the final word on a random page in either a familiar book or an unfamiliar book. Participants were asked to select one of two books for which they would be asked to solve a multiple choice question with four possible answers (**Figure 4A**). If they guessed the correct answer (25% chance), they would win an additional five tokens. The order of presentation of the books was counterbalanced across participants. Children received verbal directions and indicated their responses verbally or by pointing, while adults received written directions and indicated their response on a worksheet.

Forty-one children completed the Familiarity Bias task (one male 8.3-year-old did not complete this task due to lack of time). For children, the familiar book was always the US edition of *Harry Potter and the Sorcerer’s Stone* (HP-US) by J. K. Rowling. The unfamiliar book was either the corresponding UK edition of the same book, *Harry Potter and the Philosopher’s Stone* (HP-UK), or *Hammer of Witches* (HoW) by Shana Mlawski. For the 27 children who recognized the physical HP-US book but did not recognize the physical HP-UK book, the unfamiliar book was HP-UK. These children were told that both books were the same story about Harry Potter and his first year at magic school, but one was sold in the US while the other was sold in the UK. These children were shown and read the opening of Chapter 4 in both books in order to underscore that both books featured the same text but different page numbers.

The remaining 14 children either recognized the Harry Potter character but not the physical HP-US book or recognized both the HP-US book and the HP-UK book. For these children, the unfamiliar book was HoW. None of these children recognized HoW. These children were told that HP-US was “a story about a boy who can do magic and has adventures in his first year of magic school,” while HoW was “a story about a boy who can do magic and has adventures in his first time on a sailing ship.”

Adults did the Familiarity Bias task with HP-US and HP-UK. Thirty-two of 40 adults indicated having read either HP-US or HP-UK (12 female; mean age = 22.2 years). Their responses were coded with the edition that they had previously read serving as the familiar book. Familiarity bias data were not analyzed for the remaining eight adults who reported having read neither Harry Potter book.

## RESULTS

### BAR CHOICE

**FIGURE 2:**
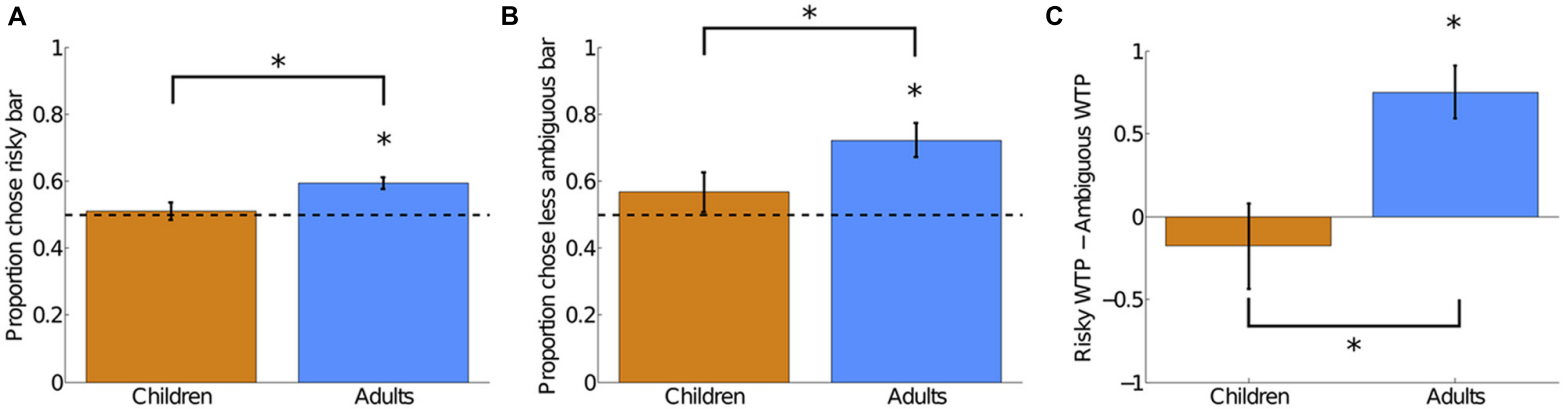
**Evidence of ambiguity aversion in adults but not in children and significantly greater ambiguity aversion in adults than in children across three separate measures. (A)** Frequency of choosing the risky bar on risky bar versus ambiguous bar trials in the Bar Choice task. **(B)** Frequency of choosing the less ambiguous bar on ambiguous bar versus ambiguous bar trials in the Bar Choice task. **(C)** Difference in willingness to pay (WTP) for risky bars and ambiguous bars in the WTP task. Dotted lines indicate chance performance. *indicates significance at *p* < 0.05.

**FIGURE 3:**
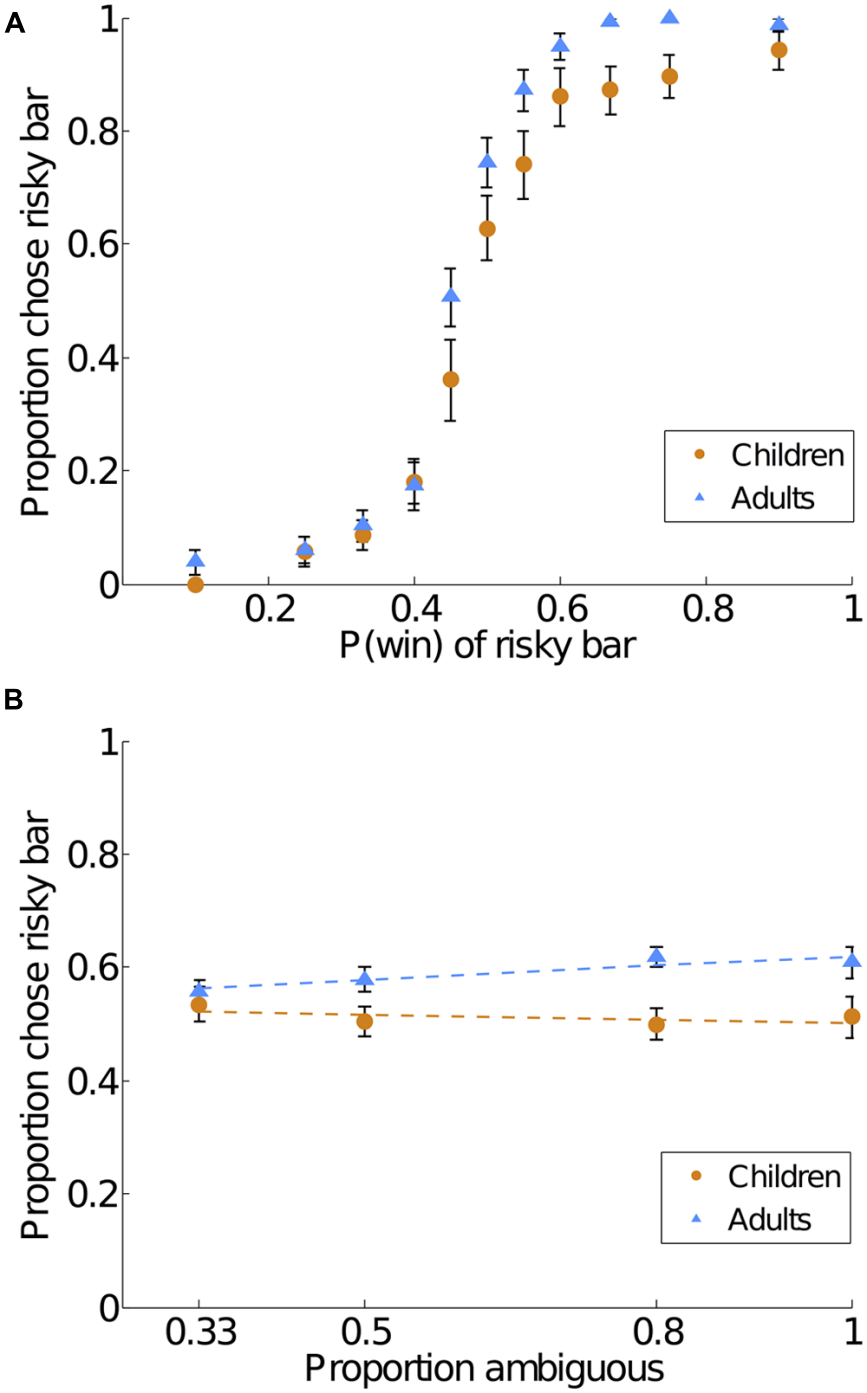
**Ambiguity aversion in children and adults at different levels of risk and amounts of ambiguity.** Note that these are the same data as **Figure 2A**, replotted here for illustrative purposes. **(A)** Frequency of choosing the risky bar on risky bar versus ambiguous bar trials, plotted by the risky bars’ probability of winning. **(B)** Frequency of choosing the risky bar on risky bar versus ambiguous bar trials, plotted by the ambiguous bars’ proportion of ambiguity. Error bars indicate SE of the mean. Dotted lines indicate linear regression fits.

On the risky versus ambiguous trials, we found evidence of (1) ambiguity aversion in adults but not in children and (2) significantly greater ambiguity aversion in adults than in children. Children chose the risky and ambiguous bars equally often on non-catch trials (chose risk 51.1% of the time; compared to 50% chance, *t*(34) = 0.41, 2-tailed *p* = 0.69). Adults chose the risky gambles 59.5% of the time on non-catch trials, significantly more often than chance [*t*(38) = 5.29, 2-tailed *p* < 0.001] and significantly more often than the children did [*t*(72) = 2.69, 2-tailed *p* = 0.01; Cohen’s *d* = 0.60; see **Figure 2A**]. These results still held when excluding the 10 children who saw only seven levels of risk [children compared to 50% chance, *t*(24) = 0.68, 2-tailed *p* = 0.51; children compared to adults *t*(62) = 2.11, 2-tailed *p* = 0.04; Cohen’s *d* = 0.53]. For illustrative purposes, we also show the data from **Figure 2A** plotted by the risky bars’ probability of winning (**Figure 3A**) and the ambiguous bars’ proportion of ambiguity (**Figure 3B**).

On the ambiguous versus ambiguous trials, we found qualitatively similar results. Children were equally likely to choose the more and less ambiguous bars [chose less ambiguous 56.67% of the time; compared to 50% chance, *t*(34) = 1.11, 2-tailed *p* = 0.28]. Adults, in contrast, chose the less ambiguous option 72.2% of the time, significantly more often than chance [*t*(38) = 4.42, 2-tailed *p* < 0.001] and significantly more often than the children did [*t*(72) = 2.00, 2-tailed *p* = 0.05, Cohen’s *d* = 0.46; see **Figure 2B**]. For both children and adults, the likelihood of choosing risky on risky versus ambiguous trials significantly correlated with the likelihood of choosing the less ambiguous bar on ambiguous versus ambiguous trials [children: *r*(33) = 0.68, *p* < 0.001; adults: *r*(37) = 0.55, *p* < 0.001], indicating consistent attitudes towards ambiguity across two different trial types within the task.

### WILLINGNESS TO PAY (WTP)

On the WTP task, we again found evidence of (1) ambiguity aversion in adults but not in children and (2) significantly greater ambiguity aversion in adults than in children. Children indicated no difference in WTP for the risky bars (average WTP = 6.51 tokens) and the ambiguous bars [average WTP = 6.68 tokens; paired *t*(33) = -0.69, 2-tailed *p* = 0.50], while adults were willing to pay significantly more for the risky bars (average WTP = 5.53 tokens) than for the ambiguous bars [average WTP = 4.78 tokens; paired *t*(38) = 4.67, 2-tailed *p* < 0.001]. The difference in WTP between the risky and ambiguous bars was significantly greater in adults than in children [*t*(71) = 3.15, 2-tailed *p* = 0.002; see **Figure 2C**].

For both children and adults, the likelihood of choosing risky on risky versus ambiguous bars in the Bar Choice task did not correlate with the difference in their WTP between the risky and ambiguous bars (all *p*s > 0.1). This result is consistent with previous findings reporting shifts in preferences when comparing forced choice and WTP tasks (Lichtenstein and Slovic, 1971).

### BAR PROBABILITY

All participants included in the Bar Choice sample reported predicted outcomes for the risky bars that correctly scaled with the bars’ actual probabilities. Children’s predicted outcomes linearly fit to the bars’ actual probability with an average slope of 1.06 (range 0.57–1.58), while adults’ predicted outcomes fit with an average slope of 1.04 (range 0.78–1.21). These results indicate that children and adults understood the meaning of the risky bar stimuli and were fairly accurate in perceiving the bars’ proportions of red and blue.

There were no significant differences in children and adults’ reported predicted outcomes for the ambiguous bars: children reported that an average of 50.6% of ambiguous bar outcomes would result in their favorable color (yield a large reward), while adults reported that an average of 51.1% of ambiguous bar outcomes would result in their favorable color [*t*(72) = -0.31, 2-tailed *p* = 0.76]. Neither group’s predicted outcomes for the ambiguous bars significantly differed from a 50% chance of resulting in the favorable color [children: *t*(34) = 0.64, 2-tailed *p* = 0.53; adults: *t*(38) = 0.88, 2-tailed *p* = 0.38]. Though both children and adults explicitly reported a rational 50–50 estimation of the ambiguous bars’ probabilities in the Bar Probability task, only children also exhibited a rational interpretation of the ambiguous bars on the Bar Choice and WTP tasks. The discrepancy between adults’ explicitly reported values on the Bar Probability task and their preferences as determined in the Bar Choice and WTP tasks was consistent with past studies finding a disconnect between true probabilities and individuals’ revealed probability weights (Tversky and Kahneman, 1992).

### FAMILIARITY BIAS

**FIGURE 4:**
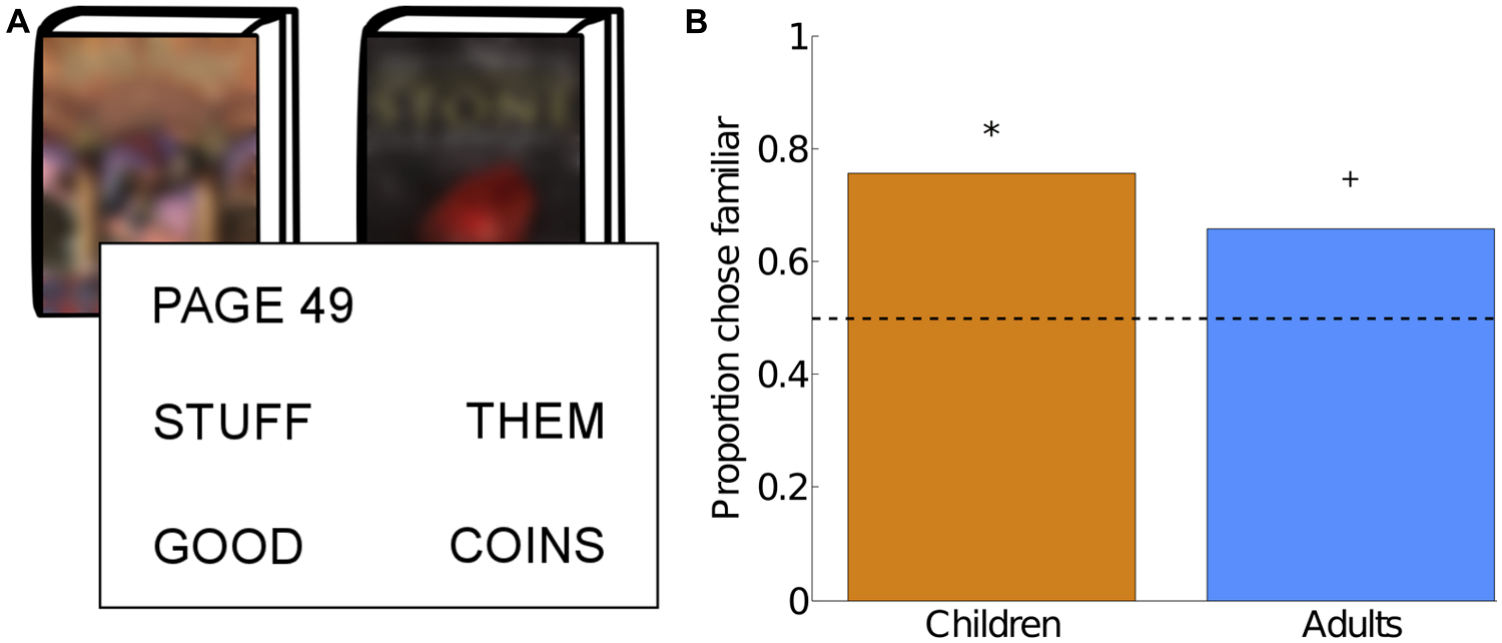
**A child-friendly guessing game found significant familiarity bias in children. (A)** Participants were asked to guess the final word on a random page of a book. Physical copies of a familiar and unfamiliar book were shown, along with a page number and four possible final words for that page. Participants first indicated if they wanted to guess for the familiar book or the unfamiliar book, then indicated their guess from the four possible final words. Correct guesses (e.g., STUFF for the familiar book and COINS for the unfamiliar book) were rewarded with five additional tokens. **(B)** Percentage of participants that chose to guess for the familiar book. Dotted line indicates chance performance. *indicates significance at *p* < 0.05; +indicates *p* = 0.11.

Children exhibited a significant familiarity bias: 75.6% of the children preferred to bet on the familiar book, a proportion significantly greater than chance (2-tailed *p* = 0.002; **Figure 4B**). This result remained significant when restricted to the 14 children who used HoW as the unfamiliar book (85.7% chose familiar, 2-tailed *p* = 0.01) and was marginally significant when restricted to the 27 children who used HP-UK as the unfamiliar book (70.4%, 2-tailed *p* = 0.05). This result also remained significant when restricted to the 34 children who both did the Familiarity Bias task and were included in the final Bar Choice sample (70.6%, 2-tailed *p* = 0.02).

Adults exhibited a marginally significant familiarity bias: 65.6% of the adults preferred to bet on the book that they reported having previously read (2-tailed *p* = 0.11; **Figure 4B**). While this is a weaker familiarity bias than what we observed in children, we note that our adult participants, all of whom lived in the US at the time of the experiment, may have recognized the physical HP-US book even if they did not report having read it. When adult analyzes were restricted to the 23 adults who reported having read HP-US, those adults exhibited a significant familiarity bias (78.3%, 2-tailed *p* = 0.01).

## DISCUSSION

Our results demonstrate that children (8–9 years old) and young adults (19–27 years old) hold significantly different attitudes towards ambiguity when making decisions about rewards. This conclusion held across two tasks and three measures. To the best of our knowledge, our study is the first to describe ambiguity preferences in pre-adolescent children.

Our study design allowed us to reject the alternative explanation that children and adults performed differently because children did not comprehend the bar stimuli. All children included in the final sample passed the same rigorous comprehension checks as the adults: (1) correctly answering comprehension questions about the risky and ambiguous bars in training, (2) correctly selecting the favorable bar on all catch trials in the Bar Choice task, and (3) reporting predicted outcomes for the risky bars that correctly scaled with the bars’ probabilities. Additionally, children, like adults, exhibited correlated ambiguity preferences during the Bar Choice task across the two different trial types (risky versus ambiguous; ambiguous versus ambiguous), demonstrating consistent attitudes towards the ambiguous bars. We also note that previous research has successfully used similar stimuli to represent probabilistic gambles with children as young as 4 and 5 years of age (Schlottmann and Anderson, 1994; Schlottmann, 2001).

We cannot, however, rule out that children were simply ignoring the occluders on the ambiguous bars and making their decisions based solely on the visibly equal amounts of red and blue on the ambiguous bars. In fact, such an approach to the ambiguous bars would represent a mathematically rational strategy that follows expected utility theory: if the midpoint-centered occluders could be covering all possible proportions of red and blue, then the average expected proportion would be a 50–50 split. Thus, simply ignoring the ambiguous occluders on the risky versus ambiguous Bar Choice trials would be a strategic, economically rational choice that does not necessarily indicate a lack of understanding of the ambiguous bars. Future studies could use ambiguous stimuli featuring visibly uneven amounts of red and blue (Peysakhovich and Karmarkar, submitted) to investigate how children use known information when assessing ambiguity.

Our study design also allowed us to probe the role of familiarity bias in causing ambiguity aversion. As adult ambiguity aversion has been theorized to result from a preference to bet on what feels more familiar (Heath and Tversky, 1991; Fox and Tversky, 1995; Fox and Weber, 2002), a natural explanation for children’s lack of ambiguity aversion could be that they are less sensitive to familiarity. Our results argue against that alternative explanation: we found that a significant familiarity bias was present in children, even though ambiguity aversion was absent. Additional studies are needed to determine if other proposed mechanisms of ambiguity aversion in adults (see Camerer and Weber, 1992 for review) are also present in children in order to determine why ambiguity aversion is absent in children. For example, a lack of ambiguity aversion can be interpreted as a sign of optimism or lack of suspicion towards the motives of the experimenter (Kuhberger and Perner, 2003), and the emergence of ambiguity aversion from childhood to adulthood may reflect an increase in pessimism or suspicion towards others that comes with life experiences. Future studies could manipulate experimenter trustworthiness (Kidd et al., 2012) in order to determine if and how children’s expectations of the experimenter interact with their attitudes towards ambiguity.

Our results are consistent with a previous study in which 12- to 17-year-old adolescents exhibited less ambiguity aversion compared to 30- to 50-year-old adults (Tymula et al., 2012). We cannot make direct comparisons between the preferences of our child sample and the adolescent sample of Tymula et al. (2012) because we used different tasks to measure ambiguity aversion. We note, however, that the adolescent sample of Tymula et al. (2012) exhibited a significant increase in ambiguity aversion as the level of ambiguity increased, while our children were indifferent to ambiguity level on the Bar Choice trials. Thus, ambiguity aversion may increase linearly with age across development, though additional studies directly comparing children, adolescents, and adults are needed to determine if this is the case. Finally, we note that our findings are restricted to decisions involving potential gains. As adolescents and adults have been found to be similarly ambiguity neutral in the loss domain (Tymula et al., 2013), future studies should compare children’s ambiguity preferences for losses to those of adults.

Our findings that children are surprisingly tolerant of ambiguity have important public health implications, as ambiguous decisions are analogous to most real-world decisions, in which outcome probabilities are not precisely known. In fact, ambiguity tolerance, but not risk tolerance, in adolescents was found to predict their engagement in real-world reckless behavior (Tymula et al., 2012). Many interventions targeted towards reducing real-world reckless behavior focus on adolescents because that age group is especially vulnerable to preventable morbidity and mortality that results from poor decision-making (Keeney and Palley, 2013). Our findings point to a developmental trajectory for ambiguity tolerance that emerges prior to adolescence, in childhood. Consequently, policies aiming to reduce reckless behavior in adolescents should consider addressing the behaviors and attitudes of children, before they grow into adolescents.

## CONCLUSION

Across three distinct measures in two different tasks, ambiguity aversion was absent in 8- and 9-year-old children but present in adults. When comparing risky gambles to ambiguous gambles, children were equally likely to choose risk or ambiguity while adults preferred risk over ambiguity. When comparing two gambles of varying levels of ambiguity, children were equally likely to choose the more or less ambiguous gamble while adults preferred the less ambiguous gamble. When assigning value to risky and ambiguous gambles, children priced them equally while adults were willing to pay more for risky gambles. We also found that children’s lack of ambiguity aversion was likely not driven by an indifference to familiarity, for children did exhibit a bias to bet on the familiar. Taken together, our results suggest that ambiguity aversion emerges from childhood to adulthood and is not caused by a bias toward familiarity.

## Conflict of Interest Statement

The authors declare that the research was conducted in the absence of any commercial or financial relationships that could be construed as a potential conflict of interest.
